# Review: Known, Emerging, and Remerging Pharyngitis Pathogens

**DOI:** 10.1093/infdis/jiae391

**Published:** 2024-10-23

**Authors:** Jane M Caldwell, Nathan A Ledeboer, Bobby L Boyanton

**Affiliations:** Medavera, Inc, Springfield, Missouri, USA; Department of Pathology and Laboratory Medicine, Medical College of Wisconsin, Milwaukee, Wisconsin, USA; Department of Pathology and Laboratory Medicine, Arkansas Children's Hospital, Little Rock, Arkansas, USA; Department of Pathology and Laboratory Medicine, University of Arkansas for Medical Sciences, Little Rock, Arkansas, USA

**Keywords:** pharyngitis, etiologic agents, emerging pathogens, reemerging pathogens

## Abstract

Pharyngitis is an inflammatory condition of the pharynx and/or tonsils commonly seen in both children and adults. Viruses and bacteria represent the most common encountered etiologic agents—yeast/fungi and parasites are infrequently implicated. Some of these are predominantly observed in unique populations (eg, immunocompromised or unvaccinated individuals). This article (part 1 of 3) summarizes the impact of acute pharyngitis on the health care system and reviews the etiologic agents of acute pharyngitis, including both emerging and reemerging pathogens that health care providers should consider when evaluating their patients. Finally, it sets the stage for parts 2 and 3, which discuss the current and evolving state of diagnostic testing for acute pharyngitis.

## CASE STUDY: PHARYNGITIS

One week into the new school year, an 8-year-old girl experiences acute onset of sore throat. Her mother notes the presence of fever (103.5°F) and a bright red throat. The following morning the patient is seen in an urgent care clinic. The health care provider confirms the aforementioned signs and symptoms as well as an abdomen that is slightly tender to palpation. Tonsillar enlargement/exudates and anterior cervical lymphadenopathy are absent. The provider, suspecting acute bacterial pharyngitis, performs a rapid antigen detection test (RADT) for group A *Streptococcus* (GAS)—the result is negative. A throat swab sample is sent to a clinical microbiology laboratory for culture-based confirmation. The mother is informed that a prescription will be provided if the culture is positive. Twenty-four hours later the laboratory notes the presence of β-hemolytic colonies suggestive of GAS embedded within normal flora. The suspicious colonies require subculturing for isolation. At forty-eight hours, isolated colonies are confirmed to be GAS using traditional microbiology methods. The provider receives the positive test result, electronically transmits the antibiotic prescription to the pharmacy of choice and notifies the mother to pick up the prescription. This process delays diagnosis and treatment, which increases the days the child is symptomatic, potentially spreading infection to others, and the risk of infection-associated complications, such as invasive head and neck infection.

This case highlights the challenges experienced by health care providers in outpatient/urgent care clinics and emergency departments that do not have access to rapid, near-patient diagnostic testing. Traditional culture-based confirmatory testing delays patient management by 24 to 48 hours. Even when considering new, state-of-the art clinical microbiology laboratories that incorporate total laboratory automation with real-time monitoring of culture growth and mass spectrometry-based pathogen identification directly from the culture plate, confirmatory testing will take a minimum of 16 hours. This approach, although promoting appropriate antibiotic utilization, negatively impacts operational efficiency of health care organizations, leads to additional school day absences, and lost wages for parents caring for ill children. Due to these testing delays, most physicians treat empirically in the outpatient setting. As such, antibiotic overuse is a much more common problem than delayed treatment or undertreatment.

## INTRODUCTION

Pharyngitis is an inflammatory condition of the pharynx and/or tonsils that is commonly seen in both adults and children and is responsible for 1%–2% of all outpatient visits in the United States annually [1–3]. GAS alone causes an estimated 5.2 million outpatient visits and 2.8 million antibiotic prescriptions annually among US persons aged 0–64 years with pharyngitis [4–6]. As one of the most common reasons for primary care visits, it is the number one diagnosis linked to antibiotic use in school-aged children [7, 8]. Individuals complaining of sore throat also frequently present to medical emergency departments. According to the National Hospital Ambulatory Medical Care Survey (2017), there were more than 1.19 million visits to US emergency departments for acute pharyngitis [9]. In one study evaluating the medical and nonmedical costs of pediatric pharyngitis, children missed a mean of 1.9 days of school or day care, and 42% of parents missed work. Further expanding the familial impact of pharyngitis, a second parent or caregiver also missed a mean of 1.5 days in 14% of families. Including indirect costs related to loss of parent's wages while missing work, the estimated total cost in the United States was calculated as between $224 and $539 million annually [10]. In this review, we will describe the key characteristics of GAS pharyngeal infections, as well as other causes of pharyngeal infections, and highlight emerging pathogens that clinicians must consider in children and adults.

## GROUP A STREPTOCOCCAL PHARYNGEAL INFECTIONS

Bacteria are the second most common etiology of acute pharyngitis. Infection with β-hemolytic GAS, accounts for 5%–15% and 20%–30% of infections in adults and children, respectively [11]. While acute pharyngitis caused by GAS can also be self-limited and resolve without antibiotic treatment, the clinical course of GAS pharyngitis can be associated with significant postinfectious sequelae and other complications [12]. Inadequately or untreated GAS pharyngitis can lead to both nonsuppurative and/or suppurative complications, especially in children 3–15 years of age [13]. Treatment consists of penicillin or amoxicillin, or for β-lactam allergic individuals, a first-generation cephalosporin, clindamycin, clarithromycin, or azithromycin [11]. Nonsuppurative complications are immune mediated and include poststreptococcal glomerulonephritis, acute rheumatic fever, and rheumatic heart disease. Suppurative complications include abscess (brain, peritonsillar region, soft tissues of head and neck), cervical lymphadenitis, mastoiditis, meningitis, otitis media, sinusitis, and rarely septicemia [13]. Based on 2010 estimates of deaths by rheumatic heart disease [14] and other previous deaths by invasive GAS infections [15], GAS was postulated to be the fifth most lethal pathogen worldwide [14, 16].

Point-of-care RADTs are routinely used to detect GAS from a throat swab sample. Negative RADT results can be confirmed by either routine or comprehensive throat culture. Routine throat culture only detects the presence or absence of GAS. Comprehensive throat culture detects the presence or absence of GAS as well as other bacterial organisms, including *Arcanobacterium haemolyticum*, β-hemolytic groups C/G streptococci, *Moraxella catarrhalis*, *Haemophilus influenzae*, etc.

Group A streptococci are β-hemolytic gram-positive cocci, typically identified by their biochemical traits (catalase-negative, pyrrolidinyl aminopeptidase positive, Lancefield group A positive), or via other resources, such as mass spectrometry or polymerase chain reaction (PCR) [15]. They contain virulence factors such as hemolysins, M-protein, and extracellular enzymes, which contribute to their invasiveness in humans [13, 15]. They are ubiquitous on human skin and mucous membranes, with humans being the only known natural host. It has been reported that 10%–25% of children with pharyngitis who culture positive for GAS are GAS carriers [17]. Another study found a high prevalence of asymptomatic carriage among adolescents and young adults without throat infections [18]. In a cross-sectional study of 217 healthy individuals between 16 and 25 years of age, samples were analyzed for GAS, groups C/G streptococci, *Fusobacterium necrophorum*, and *Arcanobacterium haemolyticum* [18]. At least 1 of those potential pathogens was detected in 25.3% (55/217) of these healthy young adults, a rate which was higher than previously described in the literature [18]. Therefore, it can be difficult to differentiate between acute infection and colonization by GAS. The clinical presentation of an individual with pharyngitis will vary according to the infectious agent. Symptoms of GAS pharyngitis include throat pain, fever, headache, and chills. Abdominal pain, nausea, vomiting, and scarlatiniform rash may also be present. Viral pharyngitis symptoms often include cough, rhinorrhea, conjunctivitis, headaches, and sometimes rash [19]. Cough, coryza, or conjunctivitis typically do not present with GAS infection [20] and favor a viral etiology. Unfortunately, symptoms of GAS may overlap significantly with viral etiologies, so obtaining a definitive diagnosis based solely on patient symptoms is difficult [21]. This often leads to misdiagnosis and misuse of antibiotic therapy.

The McIsaac score (modified Centor Score) is the most common decision rule used to clinically diagnose GAS pharyngitis and differentiate it from viral pharyngitis [22–24]. It includes a point system (0–5) for specific criteria as follows: patient age (1, 3–14 years; 0, 15–44 years; −1, ≥45 years), tonsillar swelling/exudate (1, present; 0, absent), tender/swollen anterior cervical lymph nodes (1, present; 0, absent), temperature (1, >38°C; 0, <38°C), and cough (1, absent; 0, present). Point totals are used to recommend actions: 0–1, no testing or antibiotics; 2–3, perform rapid antigen and/or culture testing; ≥4, consider rapid antigen and/or culture testing, acceptable to prescribe empiric antibiotics [22–24]. Outcome measures from a large meta-analysis (206 870 patients) found that for patients 3 years or older, 27% tested positive for GAS [25]. McIsaac scores (95% confidence interval) of positive GAS patients were tallied by percent: 8% (0); 14% (1); 23% (2); 37% (3); and 55% (4) [25]. In another study validating the guidelines for management of pharyngitis in both adults and children (n = 787), a McIsaac score of 3 or 4 was associated with a high rate of unnecessary antibiotic use when used without culture or RADTs [24]. As a priority, the medical history and clinical exam should exclude signs/symptoms consistent with complicated GAS pharyngitis, including airway obstruction and invasive disease into neck tissues [19].

## OTHER STREPTOCOCCAL PHARYNGEAL INFECTIONS

Other streptococci, specifically large-colony-forming phenotypes of β-hemolytic group C (GCS) and group G (GGS) streptococci, closely resemble GAS in their shared virulence traits and have also been implicated as causes of acute bacterial pharyngitis [13, 26]. In this group, *Streptococcus dysgalactiae* subsp. *dysgalactiae* or *S. dysgalactiae* subsp. *equisimilis* are the species most commonly associated with pharyngitis. These non-group A, β-hemolytic streptococci have caused well-documented outbreaks of pharyngitis, many of them zoonotic [26]. Consumption of nonpasteurized dairy products has also been documented as a source of GCS-induced pharyngitis [1]. Additionally, these pathogens are commonly observed in outbreaks among college students [27–29]. While these cause acute pharyngitis infections like that of GAS, neither GCS nor GGS is associated with nonsuppurative complications (poststreptococcal glomerulonephritis, acute rheumatic fever, rheumatic heart disease); therefore, the utility of antibiotic therapy to eradicate large-colony-forming streptococci has been questioned in the pursuit of appropriate antibiotic stewardship and the mitigation of antibiotic resistance [1, 13]. These large-colony-forming streptococcal phenotypes, like GAS, may colonize asymptomatic individuals. Meta-analysis and surveys have found 1.5% to 3% of children and approximately 6% of adults are group C/G *Streptococcus* carriers [26, 30].

## NON-STREPTOCOCCAL BACTERIAL PHARYNGEAL INFECTIONS

Besides streptococci, there are other bacterial pathogens that cause pharyngitis and many of these are not responsive to conventional GAS antimicrobial therapy. This drives the need for identification of additional bacterial pathogens if GAS is not detected by antigen, culture, or molecular testing modalities. Both commonly and uncommonly encountered bacterial pathogens of pharyngitis are summarized in Table 1.

**Table 1.** Etiologic Agents Associated With Acute Pharyngitis^a^

Organism	Relative Frequency^b^	Other Associated Disorders
**Bacterial (frequency 20%–50%)**		
*Streptococcus pyogenes* (group A)	**++++**	Tonsillitis, scarlet fever
*Streptococcus* (β-hemolytic, pyogenic groups C/G)^c^	**++++**	Skin and soft tissue infections, bacteremia, endocarditis, septic arthritis, osteomyelitis, puerperal infections, meningitis
*Arcanobacterium haemolyticum*	**+++**	Scarlatiniform rash
*Fusobacterium necrophorum*	**+++**	Tonsillitis, Lemierre syndrome
*Chlamydia pneumoniae*	**+**	Bronchitis, pneumonia
*Chlamydia psittaci*	**+**	Psittacosis
*Chlamydia trachomatis*	**+**	Tonsillitis, sexually transmitted infection
*Corynebacterium diphtheriae* (RP)	**+**	Diphtheria
*Corynebacterium ulcerans*	**+**	Diphtheria-like illness
*Francisella tularensis*	**+**	Tularemia, oropharyngeal form
*Haemophilus influenzae*	**+**	Ear infections, meningitis, pneumonia, sepsis, sinusitis
*Legionella pneumophila*	**+**	Legionnaire disease
*Leptospira* spp.	**+**	Leptospirosis
Mixed anaerobes^d^	**+**	Vincent angina
*Mycoplasma hominis*	**+**	Pneumonia, sexually transmitted infection
*Mycoplasma pneumoniae*	**+**	Pneumonia
*Neisseria gonorrhoeae*	**+**	Tonsillitis, sexually transmitted infection
*Neisseria meningitidis*	**+**	Meningitis, pneumonia, sepsis, septic arthritis
*Rothia denticariosa*	**+**	Endocarditis, pneumonia, tonsillitis,
*Salmonella typhi*	**+**	Typhoid fever (diarrhea and rash)
*Streptobacillus moniliformis*	**+**	Endocarditis, rat bite fever, septic arthritis, streptobacillosis
*Streptococcus agalactiae* (group B)	**+**	Neonatal disease, infectious pregnancy-related complications, tonsillitis
*Streptococcus pneumoniae*	**+**	Pneumonia
*Treponema pallidum*	**+**	Syphilis (secondary), sexually transmitted infection
*Yersinia enterocolitica*	**+**	Enterocolitis
*Yersinia pseudotuberculosis*	**+**	Mesenteric lymphadenitis (appendicitis mimic)
**Viral (frequency 50%–80%)**		
Respiratory viruses		
Adenoviruses	**++++**	Pharyngoconjunctival fever
Coronavirus (SARS-CoV-2)	**++++**	COVID-19
Coronaviruses (seasonal, not SARS-CoV-2)	**++++**	Cold
Enteroviruses^e^ (RP)	**++++**	Cold, herpangina, hand-foot-mouth disease, polio
Human metapneumovirus	**++++**	Upper and lower respiratory tract infection
Influenza A/B	**++++**	Influenza
Parainfluenza	**++++**	Cold, croup
Respiratory syncytial virus	**++++**	Bronchiolitis, cold
Rhinoviruses	**++++**	Cold
Epstein-Barr	**++**	Infectious mononucleosis
Herpes simplex	**++**	Gingivostomatitis
Cytomegalovirus	**+**	Cytomegalovirus mononucleosis
HIV	**+**	Primary HIV infection
Measles (RP)	**+**	Measles
Mpox (RP)	**+**	Rash, skin ulcer
Rotavirus	**+**	Diarrhea, emesis
Rubella (RP)	**+**	Rubella, German measles
**Fungal (frequency <1%)**		
*Candida* spp.^f^	**−**	Oral candidiasis (immunodeficiency, chronic steroid and/or antibiotic use)
**Parasitic (frequency <1%)**		
*Toxoplasma gondii*	**−**	Toxoplasmosis

^a^Noninfectious etiologies include allergies, cancer, consuming spicy foods or hot liquids, gastrointestinal reflux disease, trauma, and yelling.

^b^ **++++** most common; **+++** common; **++** less common; **+** rare; **−** primary pathogen in this category.

^c^ *S. dysgalactiae* subsp*. dysgalactiae, S. dysgalactiae* subsp*. equisimilis, S. equi, S. zooepidemicus*.

^d^Mixed anaerobes (*Prevotella* spp., *Peptostreptococcus* spp., *Fusobacterium* spp.).

^e^Enteroviruses (polio, coxsackieviruses A and B, echovirus).

^f^ *C. albicans, C. tropicalis, C. kruesi, C. guillermondii, C. lusitaniae, C. parapsilosis, C. pseudotropicalis, C. stellatoidea*.

RP, Reemergent potential (RP) organisms: *C. diphtheriae*, measles, poliovirus, rubella, due to lack of global vaccination or declining vaccination rates.

A less common cause of acute pharyngitis, *Arcanobacterium haemolyticum* is a gram-positive bacillus that morphologically resembles coryneform gram-positive rods. Individuals with *A. haemolyticum* pharyngitis exhibit symptoms that closely mimic streptococcal infection [31]. This pathogen is readily found on comprehensive throat cultures but will not be identified on a culture specifically targeting GAS. *A. haemolyticum* infection is usually found in teenagers and young adults, and infected individuals may present with a generalized rash like that of scarlet fever [1]. *A. haemolyticum*, as an agent of asymptomatic colonization, was detected in the pharynx of 1.4% of individuals (n = 217) using PCR; however, it was not detected by culture methods alone [18]. In general, this organism is susceptible to antibiotics typically prescribed for the treatment of GAS pharyngitis [32, 33]. However, due to varied reports of resistance to penicillin and macrolide antibiotics, culture and antimicrobial susceptibility testing is recommended [32–34].

Recurrent or chronic sore throat is commonly associated with bacterial species belonging to the genus *Fusobacterium*, a bacterial pathogen not identified in routine throat cultures. *Fusobacterium necrophorum* is the most common causative agent for Lemierre syndrome, an anaerobic septicemia associated with venous thrombosis in otherwise healthy, adolescents and young adults [35, 36]. Lemierre syndrome has been reported with increasing frequency since 2000 and treatment requires prolonged antibiotic therapy. Diagnosis is confirmed clinically by documenting thrombophlebitis of the internal jugular vein in conjunction with growth of the anaerobic bacterium using blood agar culture [35]. In a recent study reporting asymptomatic carriage, *F. necrophorum* was found in 3.7% and 9.2% of healthy young adults using culture and PCR methods, respectively [18]. *Fusobacterium nucleatum* is an oral commensal bacterium primarily associated with periodontal infections, abscesses throughout the human body, and adverse pregnancy outcomes (chorioamnionitis, preeclampsia, miscarriage, stillbirth, etc.). It is a less common cause of Lemierre syndrome [37]. As it is a normal inhabitant of the oral cavity, anaerobic culture and/or NAA testing modalities will readily detect this organism [37]. Health care providers must correlate test results in conjunction with the presenting signs and symptoms of the patient to help ascertain colonization versus infection with this organism.

Toxigenic *Corynebacterium diphtheriae* is the bacterial agent of pharyngeal diphtheria. Humans are reservoirs of the organism with 3% to 5% of the population deemed asymptomatic [1]. While rare, there have been cases reported in the United States (a median of 4 cases annually during 1988–1992) in nonimmunized/underimmunized persons and individuals from indigenous populations [38]. Low incidence of disease or lack of recognition of this rare disease may account for the small number of reported cases [38]. Additionally, health care disparities may exist among indigenous populations. *C. diphtheriae* and emergent toxigenic strains of *Corynebacterium ulcerans* can cause necrotic coagulum and pseudomembrane formation [1, 39]. Identifying the presence of diphtheria toxin is crucial because it is the major virulence factor causing death in those with classic respiratory diphtheria [39, 40]. Clinically, this pathogen should be suspected in a nonimmunized individual with pharyngitis and the presence of pharyngeal pseudomembranes. A clinical sample obtained from beneath the pseudomembranes should be cultured using Loeffler's or Tindale's selective media with subsequent identification of *Corynebacterium* toxin [1]. A duplex real-time PCR method using a throat swab from individuals suspected of having diphtheria was found to simultaneously detect the bacteria and toxin-encoding genes with higher sensitivity and specificity than culture [40]. Toxigenic strains of *C. ulcerans* cause bovine mastitis, but can also be transmitted to humans consuming raw, unpasteurized milk [1]. Due to the increasing popularity of raw milk consumption, this transmission modality should be considered when obtaining the patient's medical history [41]. In addition, nontoxigenic strains of *C. diphtheriae* have been observed in intravenous drug users or those who have contact with them. For this specific demographic, 4.3%–20.1% of individuals had *C. diphtheriae*-associated pharyngitis; skin and bloodstream infections were also common [42, 43].

Additional bacterial agents include *Mycoplasma pneumoniae* and *Chlamydia pneumoniae* [1]. These organisms have been detected in the throats of both symptomatic and asymptomatic individuals. In children, the *M. pneumoniae* colonization rate was noted to be 21% in the upper respiratory tract using PCR [44] and 5% in the throat using culture methods [45]. In a study investigating *Chlamydia pneumoniae* among 1108 healthy adults using both PCR and culture methods, the colonization rate was found to be 1.4% [46]. In another study, the *C. pneumoniae* colonization rate was 2% in children as compared to 0% in parents [47]. Both *M. pneumoniae* and *C. pneumoniae* are known to be primary and copathogens associated with pharyngitis, but the frequency and severity of infection by these organisms is still unclear [1].

## SEXUALLY TRANSMITTED PHARYNGEAL INFECTIONS

Sexually transmitted infections (STIs), especially *Neisseria gonorrhoeae*, should be considered in the differential diagnosis of individuals presenting with pharyngitis and a history of orogenital sex or sexual abuse [48]. Men who have sex with men (MSM) are at greatest risk for *N. gonorrhoeae* pharyngeal infections [1]. In the US, MSM at CDC surveillance sites were estimated to have experienced a 375% increase in gonorrhea incidence from 2010 to 2018 [49]. Oropharyngeal *N. gonorrhoeae* infections less commonly cause symptoms or lead to significant morbidity, but the oropharynx is thought to play a major role in the development of *N. gonorrhoeae* antimicrobial resistance *[*50]. Clinical signs and symptoms of pharyngeal gonorrhea include sore throat with an erythematous pharynx, bilateral tonsillar enlargement, a grayish-yellow exudate, and occasionally cervical lymphadenopathy, gingivitis, and glossitis [51]. Diagnosis can be facilitated by culture on modified Thayer-Martin medium [1] in conjunction with biochemical and/or mass spectrometry identification methods, or (as of the time of manuscript preparation) either of only 3 US Food and Drug Administration-approved nucleic acid amplification tests (Cepheid GeneXpert CT/NG, Roche Cobas CT/NG 6800/8800, or Hologic Aptima Combo 2). For individuals ≤14 years of age with a positive test result for *N. gonorrhoeae* and/or *Chlamydia trachomatis,* confirmatory testing should be performed using more than one identification modality to ensure test result accuracy. Such situations may signify recent and/or ongoing sexual assault or abuse of a minor and the need to enlist child protective services and local law enforcement.

*Chlamydia trachomatis* and *Treponema pallidum* should also be considered as potential sexually transmitted pharyngeal infections. In a recent study quantifying the incidence and duration of pharyngeal chlamydia among MSM, 1.4% were positive at the time of enrollment with a median duration of 6 weeks [52]. Despite the low incidence and duration of pharyngeal chlamydia relative to other sexually transmitted infections, its contribution is still relatively underreported and warrants further consideration in MSM [52]. *T. pallidum*, the agent of syphilis, can invade any organ in the body and it also facilitates the transmission of other STIs [53]. Nongenital syphilitic manifestations have become more frequent due to an overall surge in sexually transmitted infections, and *T. pallidum* has been shown to cause oropharyngeal lesions [54]. Clinicians should consider the sexual history of their patients and be aware of clinical symptoms of sexually transmitted oropharyngeal infections [55].

Herpes simplex virus (HSV) is a linear, double-stranded DNA virus that has been documented to infect virtually every mucocutaneous and visceral site in the human host [56]. HSV-associated pharyngitis is usually a clinical manifestation of primary HSV-1 infection (HSV-2 is less common), and includes malaise, myalgias, dysphagia, fever, and cervical lymphadenopathy in conjunction with exudative and/or ulcerative lesions of the posterior pharynx and tonsillar pillars [56].

## VIRAL PHARYNGEAL INFECTIONS

Viruses are the most common causes of acute pharyngitis [57–59]. In particular, respiratory viruses predominate and include adenovirus, enterovirus/rhinovirus, human coronaviruses (including severe acute respiratory syndrome coronavirus 2 [SARS-CoV-2]), influenza viruses, parainfluenza viruses, and respiratory syncytial virus [1, 57, 60]. Epstein-Barr virus and human cytomegalovirus are the most common pathogens associated with infectious mononucleosis, which is often characterized by pharyngitis, hepatosplenomegaly, and lymphadenopathy. Distinguishing between IM infectious mononucleosis-associated pharyngitis and infectious pharyngitis is difficult by clinical presentation alone and can lead to overprescription of antibiotics in 76% of presentations [61]. Individuals may be asymptomatically colonized with viruses and lead to interpretative confusion when detected using multiplex panels. Recent publications have shed insight into the asymptomatic carriage of various respiratory viruses, including SARS-CoV-2 (children 40%–45%, adults 34.9%); adenoviruses (children 10%, adults 8%); rhinoviruses/enteroviruses (children 16%, adults 5%); parainfluenza viruses (children 1%, adults 2.6%); respiratory syncytial virus (children 2%, adults 6.5%); human metapneumovirus (children 2%, adults 0%); and non–SARS-CoV-2 human coronaviruses (children 2%, adults 1%) [47, 62–64]. Diagnostic tools to correctly identify the bacterial and viral causes of pharyngitis are needed to avoid unnecessary delays in care and inappropriate antibiotic treatment.

## PATHOGENS OF REEMERGENT POTENTIAL

The increased speed and ease of regional and international travel has inadvertently facilitated the rapid dissemination of pathogens around the globe. Knowledge of reemerging pathogens is of utmost importance, especially with current trends demonstrating declining US vaccination rates. According to a recent CDC Morbidity and Mortality Weekly Report [64] national- and state-level estimates for complete vaccination of US kindergarten children for measles, mumps, and rubella vaccine (MMR), diphtheria, tetanus, and acellular pertussis vaccine (DTaP), inactivated poliovirus vaccine (IPV), and varicella (herpes zoster) have declined from 95% to only 93% coverage. This trend is concerning and decreases herd immunity for the general population, thereby placing more individuals at risk for the acquisition vaccine-preventable diseases. Herd immunity is defined as the resistance to the spread of an infectious disease within a population. It is based on the preexisting immunity of a high proportion of individuals because of previous infection or vaccination. The minimum number of immune individuals within a population needed to confer herd immunity varies by pathogen. For measles, the population herd immunity threshold is estimated to be 95% [65–67]. For rubella the threshold is 87.5% [67]. For polio, the threshold is greater than 80% [68, 69]. For diphtheria, the threshold is 75% [69]. When population immunity levels drop below these thresholds, individuals without immunity will be at increased risk of infections due to these pathogens. If vaccination rates continue to drop and dip below the minimum herd immunity threshold levels, formerly uncommonly observed pathogens will need to be strongly considered in the differential diagnosis of unvaccinated individuals presenting with pharyngitis. Of particular interest, due to their ability to cause pharyngitis, are diphtheria, measles, rubella, and poliovirus. Clinicians will need to consider a broader scope of pathogens in their diagnostic and treatment paradigms to include these vaccine-preventable infections in settings with lower vaccine uptake.

On 23 July 2022, the World Health Organization declared the mpox outbreak as a public health emergency of global significance [70]. Human mpox is genetically close to the smallpox virus and produces similar lesions [71]. Two recent meta-analyses looked at pooled prevalences of clinical features associated with mpox across 5 continents and 19 countries [70, 72]. Pharyngitis was a clinical feature in 23.0% (range, 12.7%–37.9%) [60] and 32% (range, 18%–58%) [72] of cases taken from collective data on 5472 and 5698 patients, respectively. Mpox infection should be considered in patients with pharyngitis with mpox-related features and/or a history of sexual transmission regarding this emerging and ongoing global threat [70].

## DIAGNOSTIC GAPS: POINTS TO PONDER

Pharyngitis may be caused by known pathogens such as GAS, but also by emerging and reemerging pathogens because of ever evolving geopolitical and social paradigms. To effectively diagnose and treat pharyngitis and promote antibiotic stewardship, it is imperative that new diagnostic methods keep pace. Cutting-edge technologies such as nucleic acid amplification can simultaneously detect and quantify multiple pathogens, and assist in the appropriate diagnosis, management, and outcomes of individuals with pharyngitis. However, individuals may simultaneously be colonized with bacterial and viral pathogens, which can confound the interpretation of test results and lead to unnecessary antibiotic administration or inappropriate patient management. These new detection methods must be able to distinguish between these 2 conditions while remaining cost effective. The second article of this supplement will elaborate on the current state of laboratory and point-of-care diagnostic testing for pharyngitis and address these and other knowledge gaps.

## Notes

***Author contributions.*** All authors contributed to this article.

***Financial support*.** This work was supported by QuidelOrtho.

***Supplement sponsorship.*** This article appears as part of the supplement, “Laboratory and Point-of-Care Diagnostics for Pharyngitis: Pros and Cons?,” sponsored by QuidelOrtho.
